# When a ribosomal protein grows up - the ribosome assembly path of Rps3

**DOI:** 10.15698/mic2017.05.571

**Published:** 2017-03-27

**Authors:** Brigitte Pertschy

**Affiliations:** 1 Institute of Molecular Biosciences, University of Graz, Graz, Austria.

**Keywords:** ribosomal protein, ribosomal RNA, ribosome biogenesis, chaperone, nuclear import, Rps3

## Abstract

The biogenesis of ribosomes is a central process in all dividing cells. Eukaryotic ribosomes are composed of a large 60S and a small 40S subunit, each comprising a complex assembly of ribosomal RNA (rRNA) and ribosomal proteins (r-proteins). The synthesis of these constituents is spatially separated, with r-proteins being produced by translation in the cytoplasm, while rRNA is generated by transcription in the nucleus. Hence, the arrangement of r-proteins and rRNA into large ribonucleoprotein complexes requires dedicated mechanisms ensuring their encounter in the same compartment. To this end, r-proteins need to be safely delivered to the nucleus where they assemble with the rRNA. Beyond these initial challenges, the synthesis of ribosomes does not merely comprise the joining of r-proteins with rRNA, but occurs in a complex assembly line involving multiple maturation steps, including the processing and folding of rRNA. R-proteins usually have composite rRNA binding sites, with several different rRNA helices contributing to the full interaction. Not all of these interaction sites may already be accessible at the point when an r-protein is incorporated, necessitating that some of the r-protein-rRNA contacts are formed at later maturation stages. In our two recent studies, we investigated the ribosome assembly path of r-proteins in the yeast *Saccharomyces cerevisiae* using the small subunit r-protein S3 (Rps3) as a model. Our studies revealed intricate mechanisms to protect the protein, transport it into the nucleus, integrate it into pre-ribosomal precursor particles and promote its final stable association with 40S subunits.

To familiarize you with these mechanisms, I invite you to join in with me in following the early life of Rps3 from its birth, through its cumbersome journey to the nucleus, its constricted initial allocation in pre-40S particles, until its conquest of a stable position in the 40S subunit (also shown in the model in Figure 1).

**Figure 1:**
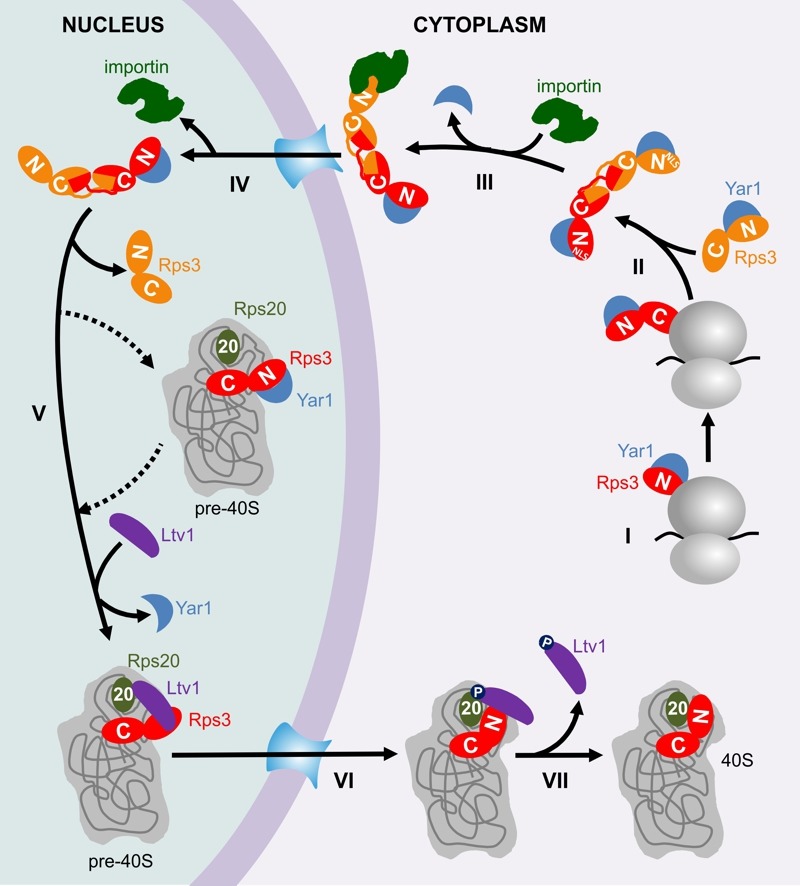
FIGURE 1: The ribosome assembly path of Rps3. **I.** The N-domain of Rps3 is co-translationally bound by the chaperone Yar1. **II. **The C-domain of Rps3 dimerizes with a second Rps3 C-domain by domain swapping. **III. **One copy of Yar1 is removed by competition with importin, which binds to the N-terminal NLS of one of the Rps3s. **IV.** The complex is transported into the nucleus, where the importin is released. **V.** One Rps3 copy is incorporated into pre-40S particles. Either after (dashed arrows) or already before Rps3 joins pre-40S, Yar1 is released and replaced by the assembly factor Ltv1. The presence of Ltv1 in pre-40S particles prevents Rps3 from attaining its final position. **VI.** Presumably after export of pre-40S particles into the cytoplasm, phosphorylation of Ltv1 and contacts between the Rps3 N-domain and ribosomal protein S20 (Rps20) lead to the release of Ltv1 and the final stable incorporation of Rps3 (VII).

Like most r-proteins, free Rps3 (outside its "natural" context in the ribosome) is prone to aggregation, presumably due to its high content in positive charges, which causes non-specific interactions with RNA 1. To prevent such undesired interactions, Rps3's delicate regions are shielded by two mechanisms. Firstly, already while it is being synthesized, Rps3's N-terminus is bound by a specific, "dedicated" chaperone, the ankyrin repeat protein Yar1 (Figure 1, Step I) 1,2. Secondly, Rps3's C-terminus dimerizes with a second Rps3 molecule by domain swapping of two beta-strands (Figure 1, Step II) 3,4.

Surprisingly, we found that *in vivo* only one of the Rps3s within the dimer is bound by Yar1 3. A potential explanation for this unexpected architecture comes from the observation that the Yar1 binding site is positioned directly adjacent to the nuclear localization signal (NLS) of Rps3 5. NLSs comprise the binding sites for importins, which mediate the transport of proteins through the nuclear pores into the nucleus 6. Our analyses suggest that several redundant routes engage in Rps3 import, with major contribution from the classical importin α/importin β pathway 5. We found that binding of importin α (Kap60 in yeast) competed with Yar1 for binding to Rps3, suggesting that one N-domain of Rps3 can only bind either importin or Yar1 (Figure 1, Step III). As we nevertheless detected complexes containing Rps3 and both Yar1 and Kap60 *in vivo*, Rps3-dimers with Yar1 bound to one and importin bound to the second Rps3 N-domain might represent the preferred conformation for nuclear import 5. It is still a puzzle how such architecture is retained, but assuming that the complex is imported immediately after one of the two Yar1 copies has been replaced by importin, the limited time for a second importin to access the second Rps3 copy might make it simply more likely that one Yar1 is maintained. After nuclear import and dissociation of importin (Figure 1, Step IV), Rps3 is incorporated into pre-40S particles, permitting that the two protective mechanisms for Rps3 transport are dismissed again by resolving Rps3 dimers and releasing Yar1 (Figure 1, Step V). Yar1 release is presumably promoted by binding of a new partner to the Rps3 N-domain, the ribosome assembly factor Ltv1 3; this may happen either before, upon, or shortly after Rps3 incorporation. It is not known how exactly Rps3 dimers are disassembled, but potentially, integration of one Rps3 sterically constraints the second Rps3, which might consequently be competed away. Although the exact positioning of Rps3 within these pre-40S particles is not known, Rps3 definitely cannot be assembled in its mature conformation, considering that Ltv1 not only associates with the Rps3 N-domain, but also occupies the rRNA site, which is entitled to the Rps3 N-domain in mature 40S subunits 3,7,8. We observed that the Rps3 N-domain alone fused to GFP displays a nuclear localization, suggesting it cannot be integrated into pre-40S particles and co-exported into the cytoplasm; consequently, the Rps3 C-domain may comprise the main anchorage of Rps3 within pre-40S particles 5.

Last but not least, after pre-40S export, Ltv1 has to be removed in order that Rps3 can move into its correct position 3. At least two different events are critical for Ltv1 release, the order of which is not yet clear (Figure 1, Step VI); (i) the formation of contacts between the Rps3 N-domain and another ribosomal protein, Rps20, which might compete Ltv1 away from one of its rRNA binding sites 3 and (ii) phosphorylation of Ltv1 by the kinase Hrr25, which presumably causes electrostatic repulsion 3,9,10. Together, these events cumulate in the release of Ltv1, allowing for the final, stable incorporation of Rps3 into 40S subunits (Figure 1, Step VII).

Together, our two studies revealed an unexpected complexity of the ribosome assembly path of Rps3. Although the details will certainly vary between different r-proteins, similar mechanisms as utilized by Rps3 may also be employed by other r-proteins. The engagement of dedicated chaperones was also reported for some other r-proteins in recent years, and more dedicated r-protein chaperones might still await their discovery 2,11,12,13,14,15,16,17,18,19,20,21.

An intriguing observation in our study was the dimerization of Rps3, which we suppose functions in protecting the C-terminal domain of Rps3 from non-specific interactions. Another r-protein, Asc1, was also reported to form dimers 22. Hence, dimerization might be a universal strategy also used by other r-proteins to shield important sites from non-specific interactions.

Last but not least, we characterized a mechanism ensuring the controlled stepwise incorporation of an r-protein by means of selective occlusion of rRNA binding sites by an assembly factor. This might provide the necessary time to sculpture the respective region before it encounters its r-protein binding partner. Subsequently, the respective assembly factor must be released in order to allow the r-protein to form its contacts with the initially withholded sites. Considering that in the course of ribosome maturation, ~250 non-ribosomal proteins bind pre-ribosomal particles at different stages in the pathway, it is likely that, analogously to Ltv1, also other assembly factors impede the full incorporation of r-proteins and that assembly factor release is connected to the stable integration of the respective r-proteins.

The investigation of the particular mechanisms utilized in the assembly paths of other r-proteins will be an interesting subject for future studies.
